# Ultrasonic Sensors in Urban Traffic Driving-Aid Systems

**DOI:** 10.3390/s110100661

**Published:** 2011-01-11

**Authors:** Luciano Alonso, Vicente Milanés, Carlos Torre-Ferrero, Jorge Godoy, Juan P. Oria, Teresa de Pedro

**Affiliations:** 1 Control Engineering Group, University of Cantabria, Avda. Los Castros s/n, 39005 Santander, Spain; E-Mails: carlos@teisa.unican.es (C.T.-F.); oria@teisa.unican.es (J.P.O.); 2 AUTOPIA Program at Center for Automation and Robotics (CAR, UPM-CSIC), 28500 Arganda del Rey, Madrid, Spain; E-Mails: vicente.milanes@car.upm-csic.es (V.M.); jorge.godoy@car.upm-csic.es (J.G.); teresa.pedro@car.upm-csic.es (T.D.P.)

**Keywords:** ultrasonic sensors, signal processing, active safety, artificial intelligence

## Abstract

Currently, vehicles are often equipped with active safety systems to reduce the risk of accidents, most of which occur in urban environments. The most prominent include Antilock Braking Systems (ABS), Traction Control and Stability Control. All these systems use different kinds of sensors to constantly monitor the conditions of the vehicle, and act in an emergency. In this paper the use of ultrasonic sensors in active safety systems for urban traffic is proposed, and the advantages and disadvantages when compared to other sensors are discussed. Adaptive Cruise Control (ACC) for urban traffic based on ultrasounds is presented as an application example. The proposed system has been implemented in a fully-automated prototype vehicle and has been tested under real traffic conditions. The results confirm the good performance of ultrasonic sensors in these systems.

## 1. Introduction

According to the Traffic Department of the Government of Spain [1], in 2009 there where a total of 88,251 traffic accidents involving deaths or injuries, and more than half of these occurred in urban areas. Although in recent years the number of victims has decreased gradually, it is still extremely high. The high number of accidents has led to the increasing use of increasingly intelligent and efficient driving-aid systems. Nowadays, autonomous vehicles driving on the roads would still be considered to be a distant possibility, but in recent years, tentative steps are being taken toward this goal. In this sense, several applications, such as Cruise Control (CC) can be found in the market [2]. Vehicles equipped with new active electronic safety systems are constantly appearing, in an attempt to reduce the risk of accidents. Most of these systems are not very efficient in preventing accidents in urban environments because the speeds and distances between vehicles are relatively low. They all use different kinds of sensors to continuously monitor the status of both the vehicle and the surrounding environment. Examples include:

- Tilt sensors, used by the control system of lights.
- High pressure sensors, used by Electronic Stability Program (ESP).
- Torque sensors, used by steering systems.
- Steering wheel angle sensors, used by steering and ESP systems.
- Acceleration sensors and seat occupation sensors, used by airbag control systems.
- Wheel rotation angle sensors, used by ESP systems.
- Wheel angular velocity sensors, used by ABS systems and their associated systems.
- Ultrasonic sensors, used by parking aid systems.
- Radar sensors, used by ACC systems.
- Vision sensors, used by new pedestrian detection systems.

All these systems increase the final price of the vehicle, and are thus are typically only offered in some high-end models. Only a few, such as ABS are usually standard in all models. Any action to reduce the cost of these systems could facilitate their integration into a larger number of vehicles.

This paper proposes the use of ultrasonic sensors in driver assistance systems, at speeds less than 50 km/h and distances less than 10 m, typical in urban traffic. These sensors can detect any obstacle within a distance range of a few tens of m, including both vehicles and pedestrians. Ultrasonic sensors are inexpensive and their hardware is simple, compared with other systems, such as those based on radar sensors or computer vision. The main disadvantages are their short range due to the attenuation of ultrasounds in air, and their slow response time due to the low propagation speed [3]. They are also greatly affected by atmospheric conditions, in particular by turbulences caused by wind and vehicle movement [4]. Despite these drawbacks, ultrasonic sensors can provide a simple and low cost alternative compared to the existing expensive and sophisticated systems, within the aforementioned speed and distance ranges, as demonstrated by experimental results obtained in this work.

Spanish law defines the safety distance as the distance enabling us to completely stop the car without hitting the vehicle in front, and allowing others to safely pass us. This distance is the sum of two factors: the driver’s reaction time and the braking distance. A system such as the one proposed can remove the driver’s reaction time, reducing the safety distance to simply the car’s braking distance, which in turn depends on several factors such as speed and road conditions.

The remainder of the paper is structured as follows: Section 2 presents the implementation of an ACC based on ultrasonic sensors, including the tuning process based on Genetic Algorithms (GA). Section 3 describes the ultrasonic sensor that has been used in this communication. An analysis of its behavior in outdoor environments is detailed in Section 4. The fully-automated vehicle and the field tests are shown in Section 5. Finally, the conclusions are presented.

## 2. Design and Tuning of the Adaptive Cruise Control

Cruise Control (CC) is a comfort system designed to maintain a constant speed preset by the driver. Its natural evolution is the Adaptive Cruise Control that can automatically slow down, decreasing the throttle when approaching a slower vehicle, and keeping a safe distance. When the preceding vehicle accelerates or leaves the path, the system accelerates once again to the preset speed. To act on the accelerator, the system uses the speed and distance to the preceding vehicle provided by a radar sensor. This system automatically turns off at the low speeds typical in urban traffic, because it cannot act on the brakes, or does so to a limited extent.

In this work an ultrasonic ACC is proposed for the speeds and distances typical in city traffic where the actual systems available on the market do not work. Unlike other existing systems, this one only uses the distance measured by the ultrasonic subsystem to control the speed and distance to the preceding vehicle, which limits its interaction with the car electronics to actions on the accelerator and brakes. This reduces the cost, and facilitates its installation in low-end vehicles. It is also capable of preventing collisions with other vehicles and pedestrians, or at least to reducing the damage or injuries.

Although the stopping distance depends approximately on the square of the speed, in [5] an ACC for urban traffic based only on the distance to the preceding vehicle provided by the sensor has been designed by simulation. To do this, a mathematical model of vehicle’s longitudinal dynamics has been used [6], implemented in Matlab/Simulink. The system estimates the relative speed and relative acceleration from consecutive samples of the distance, and uses the three quantities to calculate the actions on the accelerator and brake. In this work only the distance and relative speed are used, thus simplifying the control system.

**Figure 1.**
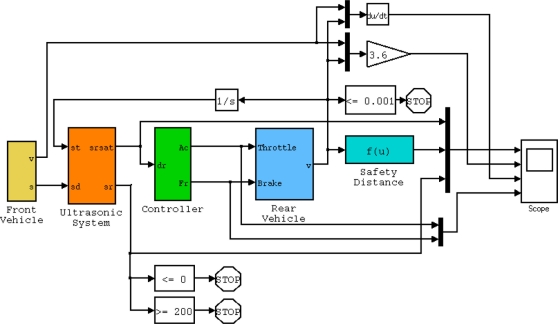
Matlab/Simulink block diagram used to perform simulations.

Figure 1 shows the block diagram used for the simulations, in which the following subsystems can be observed:

- Front vehicle, which provides the speed and distance traveled by the vehicle ahead.
- Ultrasonic system, which measures the distance between vehicles 10 times per second, limited to between 1 and 10 m due to the limitations of the sensor.
- Controller, which calculates the actions on the accelerator and brake. The output values are between 0 and 100 as a percentage of total capacity.
- Rear vehicle, which incorporates the aforementioned mathematical model of the vehicle’s longitudinal dynamics.
- Safety Distance, which calculates the braking distance based on vehicle speed according to the expression:
  (1) $$d_{b}=\frac{v^{2}}{2\cdot \mu \cdot g}$$
  with *d_b_* being the braking distance, *v* the vehicle speed, *μ* the friction coefficient between tyres and road, and *g* the acceleration of gravity. This expression is derived from the equations of uniformly accelerated linear movement [5], and at low speeds it is a very good approximation to the values obtained from simulations carried out with the mathematical model.

The control system calculates the actions on the accelerator and brake according to the following equations:

(2) $$\begin{array}{l} v=k_{1}\cdot d+k_{2}\cdot v_{r}-k_{3}/(d-1)\\ \mathrm{ac}=\{\begin{array}{ll} v & v>0\\ 0 & v\leq 0 \end{array}\;\;\mathrm{fr}=\{\begin{array}{cc} -v & v<0\\ 0 & v\geq 0 \end{array} \end{array}$$

where *ac* is the action on the accelerator and *fr* the action on the brake, *d* the distance between vehicles (limited to between 1 and 10 m.) and *v_r_* the relative speed estimated from consecutive samples of the distance. As can be seen, it is a nonlinear equation system whose first term tends to accelerate the vehicle when the distance increases, the second term tends to accelerate when the difference in the vehicles speeds increases, and the third term tends to prevent the vehicles getting too close.

**Figure 2.**
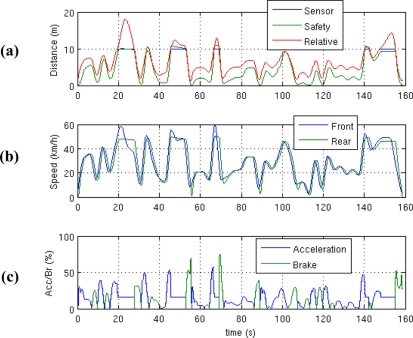
Result of a simulation with the controller obtained by the Genetic Algorithm. **(a)** Distance between vehicles, distance measured with the ultrasonic sensor (between 1 and 10 m), and braking distance. **(b)** Speed of both vehicles. **(c)** Actions on accelerator and brake.

Parameters *k_1_*, *k_2_* and *k_3_* were obtained through an optimization algorithm aimed at minimizing the following cost function:

(3) $$J=\frac{w_{1}}{T}\int_{0}^{T}{\left[d-\left(d_{b}+1\right)\right]}^{2}\mathrm{dt}+\frac{w_{2}}{T}\int_{0}^{T}\left(d<d_{b}\right)\;\left(d_{b}-d\right)\;\mathrm{dt}+\frac{w_{3}}{T}\int_{0}^{T}\left(v_{r}>50\right)\;\left(v_{r}-50\right)\;\mathrm{dt}+\frac{w_{4}(300-T)}{T}$$

where *J* is the cost, *T* is the total time simulated, *d* is the distance between vehicles, *d_b_* is the braking distance at the current speed, and *v_r_* is the relative speed between vehicles. All the *w_i_* weights assigned to each term are assumed to be equal to 0.25. The first term penalizes the difference between the following distance and braking distance plus one metre for safety. The second term penalizes the distances below the braking distance. The third term penalizes speeds of the controlled vehicle above 50 km/h, which is the typical legal urban speed limit in Spain. The last term penalizes simulations that end too soon, which means that the controlled vehicle has not been able to follow the vehicle in front during the whole simulation. As can be seen, the cost J of a controller is an unknown function of *k_i_*. The cost value depends on the result of a simulation performed with that controller, but not directly on the parameters, and is neither continuous nor differentiable, so that traditional methods are not suitable for its optimization. In this work a GA [7] was used for this task, although any other global optimization method valid for this kind of problems could have been used, as the *Simulated Annealing* method. GA’s are a well-known heuristic method for the global optimization of arbitrarily complex functions with one or more variables, based on the genetic evolution of species. It is therefore considered unnecessary to describe the details of its implementation in this work. The objective function of a GA is dependent on the problem, and there is no design methodology, except trial and error. In this work several cost functions have been tested trying to optimize the distance between vehicles, subject to the restrictions described, all with similar results, *i.e.*, obtaining very similar controllers. The proposed function is only one of them. The parameters of the GA are set so that the execution of the algorithm several times has led in all cases solutions sufficiently close together to be able to say with some confidence that these solutions are in the vicinity of global optimum. The best values obtained by the GA for the parameters are *k_1_* = 1.77, *k_2_* = 12.14 and *k_3_* = 15.75, and the final cost is *J* = 0.63. Figure 2 shows the results of a 160-s simulation with the controller obtained, during which the leading vehicle varies its speed randomly between 0 and 60 km/h.

As shown in Figure 2(a), the distance between vehicles is always greater than the stopping distance, even though the second is not known by the control system, because it does not know the absolute speed. Figure 2(b) shows that the controlled vehicle never exceeds 50 km/h. even though the vehicle ahead does so. When the distance between vehicles exceeds 10 m, which is beyond the sensor range, the controlled vehicle stabilizes its speed at about 50 km/h. In Figure 2(c), the control actions on the accelerator and brake are shown. At the end of the simulation, it can be seen how the leading vehicle performs a sudden braking maneuver, and the controlled vehicle stops at a distance of slightly more than one metre from it without crashing, the brakes remaining at a value around 30% of their capacity.

As a conclusion, we can say that a control system based solely on the distance measured by the sensor (limited to between 1 and 10 m) is able to adjust the distance, maintaining it greater than the stopping distance despite not knowing the absolute speed. However, it is necessary to have a mathematical model of the vehicle that enables the control system to be tuned prior to its experimental implementation.

## 3. Ultrasonic System

**Figure 3.**
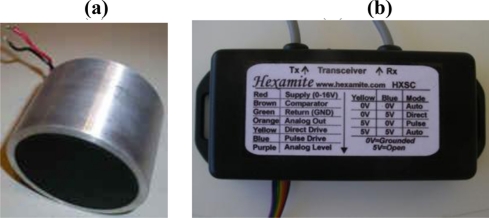
**(a)** Ultrasonic sensor. **(b)** Signal conditioner.

A prototype has been implemented using a Hexamite HXN43TR ultrasonic sensor acting as transmitter-receiver, shown in Figure 3 together with its signal conditioner. The most important characteristics of this sensor are its central frequency of 43.0 ± 5.0 kHz, its bandwidth of 4 kHz and its narrow beam pattern of 8.5° at −3 dB.

To trigger the ultrasonic sensor and to capture and process the signal received, a laptop equipped with a data acquisition card has been used. Signal processing is performed ten times per second, according to the model described in the previous section. This processing involves several stages:

1. Filtering to reduce the noise level through a band-pass filter with lower and upper cutoff frequencies of 42 and 44 kHz respectively.
2. Calculation of the Hilbert transform to obtain the envelope of the signal.
3. Raising the signal envelope to the third power, to selectively amplify those parts with greater amplitude. This improves the discrimination between the signal and the noise or the false echoes caused by turbulences or irregularities in the road.
4. Amplification with an exponential gain to compensate for the attenuation of the ultrasonic wave.

**Figure 4.**
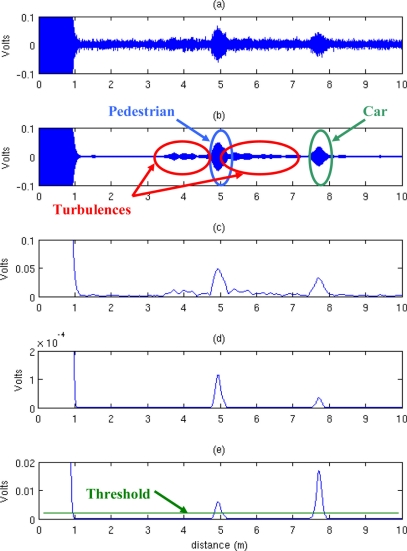
**(a)** Ultrasonic signal received by the sensor. **(b)** Filtered signal. **(c)** Envelope. **(d)** Envelope raised to the third power. **(e)** Resulting amplified signal and threshold level.

Finally, the distance between vehicles is obtained by comparing the resulting signal with a preset threshold level. The first time the signal exceeds this threshold is the well-known time of flight. The distance is calculated by multiplying the time of flight by the speed of sound in air, and halving to take into account that the ultrasonic pulse goes back and forth between the sensor and the vehicle ahead. Figure 4 shows a real ultrasonic signal acquired with the ultrasonic sensor described, together with the results of the processing steps. The capture time is equivalent to a maximum distance of 10 m. The emitted pulse is about 1 m long, during which it is not possible to receive any echo. For this reason the output of the mathematical model of the ultrasonic system was bounded between these two values. Echoes from a pedestrian located at 4.8 m from the sensor and a vehicle located at 7.5 m can be observed. The effects of turbulences can be seen in Figure 4(b,c), while in Figure 4(d,e) they are not visible.

## 4. Analysis of Errors

An insufficiently high threshold level causes the detection of false echoes produced by turbulences and irregularities in the road, making the control system act on the brakes unnecessarily. In contrast, if the threshold is too high detection failures can occur, meaning that no objects are detected when they are there and therefore causing possible collisions.

**Figure 5.**
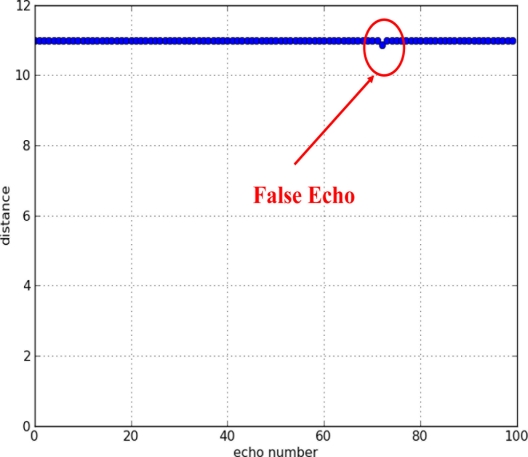
Measurements made with the sensor at rest and no obstacles ahead.

To adjust the threshold level, 100 echoes were recorded in a range of 11 m, with the sensor at rest and no obstacles in front. The threshold has been reduced slowly, until at a final value of 2 × 10^−4^ a false echo has been detected. Figure 5 shows the measurements resulting from this experiment.

**Figure 6.**
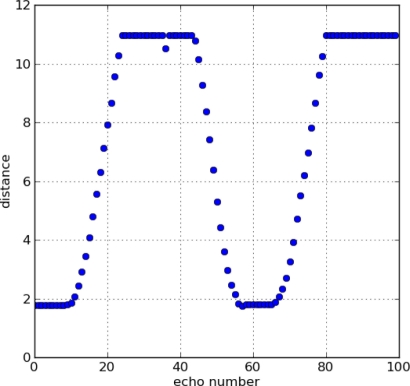
Measurements made with the sensor placed in a car moving in front of a parked vehicle.

Thereafter, this threshold level has been used in a second experiment, with the sensor placed in a vehicle which approaches and moves away from another vehicle at rest. During the maneuver, the distance was measured 100 times, showing the results in Figure 6. The distance was varied from about 2 m to more than 11, showing only one false echo that occurred when the distance between vehicles was higher than the range of the ultrasonic system. There have been no detection failures, *i.e*., when the vehicle was within the range of the sensor, it was always correctly detected.

**Figure 7.**
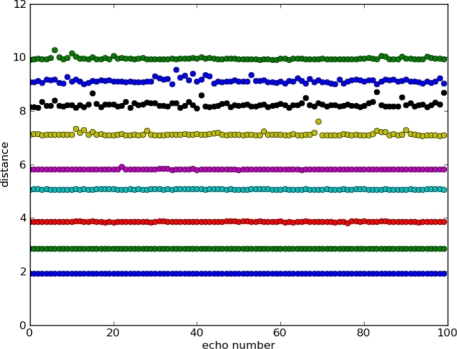
Measured distances from the sensor to a parked vehicle.

In a third experiment, the distance from the sensor to a vehicle while both were at rest, was measured 100 times at several distances from 2 and 10 m, and the results are shown in Figure 7.

It can be seen how the measurement dispersion increases with distance, because of the increasing effect of turbulence. For each distance, the median of the 100 measurements was considered as the correct distance, and the standard deviation and the maximum relative error were calculated. The results are shown in Table 1.

As can be seen from the table, both the standard deviation and the maximum relative error increases with distance, because it increases the effect of turbulence producing variations in the speed of sound. Despite this, the maximum relative error was 7% for a distance of 7 m. It should be noted that there were no false echoes with the threshold level set in the first experiment, but they might occur with an increase in the intensity of the turbulences. Increasing the threshold to avoid this produces a greater number of detection failures. Despite the number of detection failures, the probability of two consecutive occurrences is very small, and because of the short time interval between measurements, the effect of a failure is filtered by the dynamics of the vehicle, as discussed below.

## 5. Experimental Results

**Figure 8.**
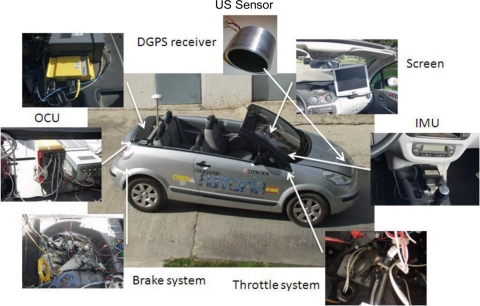
Experimental prototype.

The ultrasonic system described above was installed in a prototype vehicle, which is part of the AUTOPIA program autonomous vehicle fleet [8]. Specifically, a convertible Citroën C3 Pluriel has been used [9] for this application. The vehicle is a commercial car with the minimum modifications to manage the actuators autonomously. Figure 8 shows the vehicle and the different systems that have been installed during its automation process. Hereafter, vehicle modifications are described.

Regarding the throttle pedal, a potentiometer converts the pressure applied on the pedal by the driver into an analog signal that can be read by the vehicle’s control unit. For its automation, the signal coming from the potentiometer was cut and a switch was installed in order to choose between the analog signal coming from the potentiometer (via the throttle pedal) or the signal coming from an analog output card connected to the On-board Control Unit (OCU) which is controlled via software.

With respect to the brake pedal, the automation process was carried out with a single pre-requisite. The autonomous braking system cannot deactivate the original braking circuit. Bearing this in mind, a shuttle valve was installed before the car’s ABS. This valve (with two inputs and one output) permits the brake fluid circulation depending on the maximum pressure applied to its inputs. This valve carries out the same operation as the switch installed in the throttle pedal but, in this case, it is possible to act both on the autonomous system and the original one simultaneously. To generate the pressure to be applied, a pump was installed in the trunk of the vehicle with three additional valves. These valves are use to regulate the pressure exerted on the input of the shuttle valve. They are controlled by an analog card connected to the OCU. The control signals to manage the analog car are generated via software [10].

Different sensors are installed in the vehicle to check different automotive applications. In this case, we have only used the sensors to obtain the vehicle’s positioning in order to compare it with the distance obtained via the ultrasonic sensor. The vehicle’s location is found via a sensorial fusion of a Differential Global Positioning System (DGPS), an Inertial Measurement Unit (IMU) and the distance cover by the car obtained via the Controller Area Network (CAN) bus [11]. Finally, for comparison with the values obtained via the ultrasonic sensor, the vehicle’s speed is also obtained via the CAN bus. In previous work, a Hall-effect sensor was installed to obtain the greatest possible precision in the speed measurement [12] but, for this application, the resolution obtained from the CAN bus is enough.

**Figure 9.**
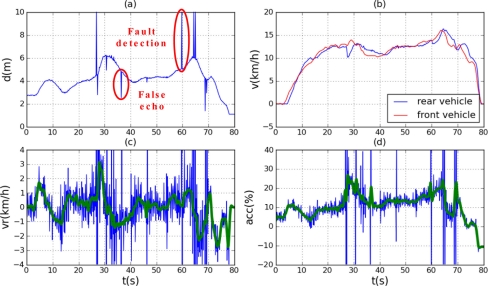
Results of a tracking maneuver performed with the proposed control system. **(a)** Distance measured with the ultrasonic system. **(b)** Vehicle speeds extracted from the CAN bus. **(c)** Relative speed unfiltered and filtered. **(d)** Actuating signal unfiltered and filtered.

The controller parameters obtained by the GA have been manually adjusted for safety, to slightly reduce the action on the accelerator, because the mathematical model does not match the vehicle used for testing, for which no model was available. Several tracking tests were performed with satisfactory results. Figure 9 shows the result of one 80-second test. Figure 9(a) shows the distance between vehicles as measured by the ultrasonic system, where detection failures and false echoes caused by turbulences can be seen. Figure 9(b) shows the speeds of the vehicles, both starting from rest at a distance of about 3 m, and both ending up at rest at a distance slightly greater than 1 m. Figure 9(c) shows the relative speed estimated by the control system from consecutive samples of the distance, along with the corresponding filtered signal. Figure 9(d) shows the control signal which acts on the throttle when it is positive or on the brake when it is negative, along with the corresponding filtered signal. Initially, the controller acts at 5% on the throttle, which is not enough to initiate the movement. The final action is about 10% on the brake, which keeps the vehicle at rest.

Unfiltered distance and unfiltered relative speed have been used as inputs to the control system. The method of estimating the relative speed makes it a very noisy signal, especially at long distances where measurement dispersion increases. The noise in the relative speed produces a noisy action. However, the speed graph shows that the speed of the rear vehicle varies smoothly, indicating that the dynamic behavior of the vehicle filters the actuating signal.

At 27 s, there is a false echo following a detection failure, caused by a gust of wind. This has produced large positive and negative peaks in the actuating signal, although for a very short duration. The consequence of these peaks is a slight braking in the speed curve of the controlled vehicle. The braking effect of false echoes is greater than the accelerating effect of detection failures, due to the different dynamics of the vehicle during acceleration and braking.

In conclusion, it can be seen how the controlled vehicle adjusts its speed to that of the vehicle ahead, and how the distance varies depending on the speed.

## 6. Conclusions

In the present work, a prototype of an ultrasonic ACC for low speeds and short distances typical of urban traffic, where the systems available on the market do not work, has been designed, built and experimentally tested. An ultrasonic sensor, cheaper and less demanding of hardware than other types of sensors currently used, such as those based on radar or computer vision, is used to measure the distance between vehicles. The relative speed is estimated using consecutive samples of this distance. These two quantities are used by the control system to calculate the actions on both the accelerator and the brake, and to adjust the speed in order to maintain the safety distance. As ultrasonic sensors can detect any kind of obstacle, this system can also prevent collision with pedestrians, or at least reduce the injuries sustained.

Since the control system does not use the absolute speed to calculate the safety distance as do the currently existing systems, interaction with automotive electronics is limited to actions on the accelerator and brake. This fact, coupled with the lower cost of ultrasonic sensors compared with other kinds of sensors, could facilitate the mounting of the system in low-end vehicles, helping to improve comfort and safety at a reduced cost.

## Figures and Tables

**Table 1.** Results of measurement of distances between the sensor and a parked vehicle.

**Median**	1.928	2.851	3.867	5.075	5.819	7.114	8.215	9.113	9.949
**Std.**	0.002	0.002	0.011	0.005	0.011	0.068	0.112	0.085	0.047
**Max. Rel. Error**	0.0026	0.0022	0.0112	0.0036	0.0159	0.0701	0.0591	0.0468	0.0323
